# *Mycoplasma pneumoniae* pneumonia with and without viral pathogens in preschool-aged children: a comparative study of clinical outcomes

**DOI:** 10.3389/fped.2026.1744105

**Published:** 2026-06-24

**Authors:** Qiuyue Yan, Hao Bi, Li Dai, Mengxin Shen, Zuliang Shi

**Affiliations:** 1 Department of Laboratory Medicine, Maternal and Child Health Hospital of Hubei Province, Tongji Medical College, Huazhong University of Science and Technology, Wuhan, China; 2 Department of Pediatric Respiratory Medicine, Maternal and Child Health Hospital of Hubei Province, Tongji Medical College, Huazhong University of Science and Technology, Wuhan, China

**Keywords:** *mycoplasma pneumoniae* pneumonia, viral co-infection, age-specific patterns, pathogen-specific phenotypes, human metapneumovirus (HMPV), disease severity

## Abstract

**Background:**

Viral co-infections are common in pediatric *Mycoplasma pneumoniae* pneumonia (MPP), yet their age-specific clinical impact on children under six is not fully understood. We aimed to describe the frequency, pathogen-specific inflammatory signatures, and age-dependent clinical impact of viral co-detection in this population.

**Methods:**

In this retrospective study, we collected data from the medical records of 504 children hospitalized with MPP between July 2021 and December 2024. The information extracted included clinical manifestations, laboratory findings, treatment modalities, and the presence of viral co-infections.

**Results:**

Viral co-detection occurred in 193 (38.3%) children, peaking in the 2–4 years age group (47.1%). Among children under 2 years, co-detection was associated with a higher severe disease rate (29.7% vs. 9.5%; *P* = 0.046). Compared to rhinovirus (HRV) co-detection, human metapneumovirus (HMPV) and respiratory syncytial virus (RSV) co-detections were linked to more pronounced inflammatory responses. HMPV co-detection was characterized by leukopenia [median: 2.25 (IQR 1.73–3.05) vs. 2.70 (1.72–7.31) × 10⁹/L, *P* = 0.013] and elevated IL-10 [8.76 (5.58–18.66) vs. 5.00 (2.97–5.95) pg/mL, *P* = 0.037] and IFN-*γ* [4.34 (2.68–5.96) vs. 2.24 (1.12–2.77) pg/mL, *P* = 0.028]. RSV co-detection featured a neutrophilic response, with higher neutrophil counts [5.76 (4.11–7.36) vs. 3.93 (2.69–4.06) × 10⁹/L, *P* = 0.033], neutrophil-to-lymphocyte ratio [2.87 (1.57–4.30) vs. 1.17 (0.62–2.13), *P* = 0.003], and IL-10 levels [10.97 (7.74–20.23) vs. 5.00 (2.97–5.95) pg/mL, *P* = 0.024]. Multivariable analysis identified wheezing (aOR=1.909; 95% CI: 1.131–3.222), severe pneumonia (aOR=1.797; 95% CI:1.019–3.169), and higher serum IL-10 (aOR=1.038 per unit; 95% CI: 1.015–1.062) as independent risk factors associated with viral co-detection. In contrast, children aged 4–6 years had a significantly lower risk of co-detection compared to those under 2 years (aOR=0.528; 95% CI: 0.308–0.903) (all *P* < 0.05).

**Conclusion:**

Viral co-detection in preschool children with MPP is associated with more severe clinical outcomes, particularly in those under two years of age. Our findings identify pathogen-specific immune response patterns (driven by HMPV and RSV) and elevated serum IL-10 as key risk markers. These findings are hypothesis-generating; the observed associations do not establish causality, and require validation in prospective studies before informing clinical practice.

## 1 Introduction

*Mycoplasma pneumoniae* (MP) is a predominant causative pathogen for respiratory infections in children. Preschool and school-aged children are vulnerable to their infections, and most cases involve those older than 5 years (1–3). MP infections occur year-round and typically have a subacute onset characterized by symptoms such as fever, cough, wheezing, and dyspnea (4–6). A recent report indicated an overall increase in the prevalence of MP from 8.12% to 14.94% among Chinese children (predominantly school-aged) from 2022 to 2023 (7). Preschool children represent a high-risk population for MP infection. Research indicates that infections caused by mycoplasma pneumoniae tend to be more severe in preschool-aged children (8). Children younger than 5 years have a high risk of Mycoplasma pneumoniae pneumonia (MPP) with viral co-infection due to their immature immune systems and weaker respiratory barrier function (9, 10).

Epidemiological analyses revealed that children with community-acquired pneumonia (CAP) and co-infection by respiratory viruses [such as respiratory syncytial virus (RSV), influenza virus, and adenovirus] had worse disease and radiological findings (such as pulmonary consolidation and pleural effusion) than those with single infections (11). Timely identification of co-infecting viral pathogens may help guide antiviral use where indicated, as evidence suggests that early initiation of neuraminidase inhibitors is associated with reduced disease severity in influenza-complicated pneumonia (12).

Although macrolides are widely recommended as first-line therapy for MPP in children based on current guidelines, their ability to meaningfully shorten disease course or reduce symptom duration remains debated. A meta-analysis by Biondi et al. found no significant benefit of macrolides over comparator treatments in terms of clinical cure rate (13), underscoring the need for continued evidence-based reassessment of treatment approaches, particularly in the context of rising macrolide resistance (13). Viral co-infections (such as by adenovirus) and antibiotic resistance are potential risk factors for worse pneumonia (14). The spectrum of respiratory pathogens co-detected with MPP may vary across different regions in China due to the vast geographical expanse. There are limited reports on co-infecting viruses and their clinical impact in preschool-aged children, particularly in the context of regional variability in pathogen prevalence.

The aim of this study was to provide a scientific basis for the prevention and treatment of MPP in preschool children by investigating the profiles of respiratory viruses responsible for co-infections in children with MPP in the Wuhan region and comparing them across age strata and viral co-infection types.

## 2 Materials and methods

### 2.1 Patients and groups

The current study involved a retrospective analysis of electronic health records from the Maternal and Child Health Hospital of Hubei Province. Data was obtained from the Jiedaokou, Hongshan, and Optics Valley campuses between July 2021 and December 2024. This study was approved by the Review Committee of the Maternal and Child Health Hospital of Hubei Province (2025-149-01) and follows Good Clinical Practice (GCP) and the Helsinki Declaration to protect participants’ health and rights. The incidence rate of MP exhibits significant variation across different age groups. School-aged children over the age of 6 years are at a heightened risk of infection; however, children aged 0 to 6 years also face considerable risk, with distinct stratified characteristics observed within this population. Notably, those under the age of 2 years are classified as being in the low-risk group (15). Furthermore, given the limited availability of research data concerning preschool children and the substantial amount of such data available at our hospital, this study focuses on children under six years old as research subjects. These subjects are further categorized into three developmental stages: infancy (under 2 years), toddlerhood (ages 2 to 4), and preschool age (ages 4 to 6). This classification is based on the observation that, in clinical practice, children aged 0 to 2 years tend to show a greater susceptibility to severe conditions and co-infections with other pathogens (16, 17). The group of children aged 4 to 6 years may have a heightened ability to recognize the early characteristics of preschoolers with MPP. By categorizing age groups in this way, we hope to clarify patterns of infection, risk factors, and treatment responses. Five hundred and four patients with MPP who were younger than 6 years were ultimately enrolled in this study based on the inclusion and exclusion criteria. Polymerase chain reaction testing was the standard procedure for all patients admitted for respiratory tract infections at our institution.

### 2.2 Diagnosis and definitions

The diagnostic criteria for MPP were based on the Chinese expert consensus on the diagnosis and treatment of Mycoplasma pneumoniae pneumonia in children (version 2023) (18): They were (a) respiratory symptoms such as fever, cough, wheezing, dyspnea, and pulmonary rales; (b) typical chest imaging changes (interstitial infiltration, segmental, and lobar consolidation); and (c) a positive result for MP-DNA detected by nucleic acid amplification tests from throat swab specimens. Severe MPP was diagnosed in patients who initially met the general diagnostic criteria for MPP and subsequently displayed signs of severe pneumonia as defined by established guidelines (19), which is consistent with the criteria used in previous studies (11). Key indicators of severity included systemic toxicity (e.g., altered consciousness, persistent high fever), respiratory distress (e.g., difficulty breathing, hypoxemia with SpO₂ < 93%), or significant radiological findings (e.g., extensive pulmonary infiltration, pleural effusion, necrotizing pneumonia). The exclusion criteria were as follows: (a) age of ≤ 1 month or > 6 years; (b) underlying diseases such as immune deficiencies, chronic respiratory diseases (such as congenital ciliary dyskinesia, diffuse interstitial lung disease, bronchopulmonary dysplasia, and bronchial asthma), congenital heart diseases, diabetes, Down syndrome, and epilepsy to avoid analytic bias regarding disease severity; (c) bacteremia; (d) an interval of less than 2 months between two hospitalizations; and (e) in addition to viral infections, other pathogenic infections were also identified or incomplete clinical data. Extrapulmonary manifestations included central nervous system involvement (encephalopathy or pleocytosis in cerebrospinal fluid analysis), elevated liver enzyme concentrations (above 80 U/L for aspartate transaminase or alanine transaminase), and dermatological involvement. Delayed treatment referred to the lack of appropriate treatment or hospitalization within 5 days (< 120 h) after fever onset (20). Fever was defined as an axillary temperature ≥ 37.5 °C (21), and measured using a calibrated electronic thermometer.

### 2.3 Data collection

The demographic and clinical information of the pediatric cohort included age, gender, duration of hospitalization, duration of cough and fever symptoms, peak recorded temperature, severity of illness, presence of extrapulmonary complications, and clinical manifestations such as vomiting, diarrhea, chills, wheezing, lung auscultation findings and lymphadenopathy. The treatment data included macrolide use, concomitant antibiotic administration, systemic corticosteroid therapy, bronchoalveolar lavage procedures. The inflammatory biomarkers included cytokine profiles, specifically interleukin (IL)-10, tumor necrosis factor-alpha (TNF-α), interferon-gamma (IFN-*γ*), and hematological parameters [leukocyte count, neutrophil count, lymphocyte count, and C-reactive protein (CRP) concentration].

### 2.4 Microbiological and laboratory examination data

Multiplex reverse transcription polymerase chain reaction (PCR) (ABI QuantStudio Q5, USA) was used to test nasopharyngeal swab samples for 13 respiratory pathogens: human metapneumovirus (hMPV), adenovirus(ADV), bocavirus (Boca), respiratory syncytial virus (RSV), coronavirus, parainfluenza virus (PIV), influenza virus [influenza A (H1N1, H3N2) and influenza B], human rhinovirus (HRV), *Mycoplasma pneumoniae* (MP), and *Chlamydia pneumoniae*. Pre-hospitalization blood samples were analyzed using the Mindray BC5310 hematology analyzer (China) for complete blood count and CRP quantification. For cytokine analysis, 3 mL of blood samples were centrifuged at 1,000 × g for 20 min. Serum samples were collected, and the concentrations of Th1 and Th2 cytokines (IL-2, IL-4, IL-6, IL-10, TNF-α and IFN-*γ*) were determined using the FACScalibur™ flow cytometer (BD Biosciences, USA) with the BD™ CBA Human Th1/Th2 Cytokine Kit II. The detection range for all cytokines was 1–5,000 pg/mL. All samples, including 13 respiratory pathogens tests and cytokine assays, were kindly collected on the first day of hospitalization. Blood routine specimens were either obtained and analyzed during the outpatient visit prior to hospitalization or on the first day of admission.

### 2.5 Routine testing practices

At our institution, MPP can be diagnosed through multiplex RT-PCR panel (assaying 13 respiratory pathogens including MP), standalone MP-targeted PCR, or MP serology, at the discretion of the attending clinician based on clinical presentation. The present study was restricted to children diagnosed via the multiplex panel pathway, because only this pathway generates concurrent data on MP status and viral co-detection. Serum Th1/Th2 cytokine profiling (IL-6, IL-10, TNF-α, IFN-γ) is performed as part of standardized admission workup for children with pneumonia at our institution and was available for all 504 children in the present cohort. We acknowledge that routine cytokine profiling is uncommon in general pediatric pneumonia practice internationally; our cytokine findings should therefore be interpreted as descriptive observations rather than a recommendation for broader clinical adoption. Bronchoalveolar lavage was performed in selected patients based on clinical indication, primarily for therapeutic purposes (airway clearance in cases of lobar atelectasis or mucus plugging refractory to medical management) and secondarily for diagnostic purposes (pathogen identification when non-invasive testing was inconclusive).

### 2.6 Statistical analysis

All analyses were performed using IBM SPSS Statistics version 25.0 with two-sided α = 0.05. Continuous variables are presented as median (IQR) and compared using the Mann-Whitney U test, as most variables departed from normality on visual inspection and Shapiro-Wilk testing. Categorical variables are presented as frequencies (percentages) and were compared using Pearson's χ² or Fisher's exact test.

Multivariable logistic regression was used to identify factors independently associated with viral co-detection. Candidate variables were selected based on univariate *P* < 0.10 and a priori clinical relevance, excluding variables that were statistically or biologically redundant. Among the three elevated pro-inflammatory cytokines (IL-10, TNF-α, IFN-γ), IL-10 was retained as the representative cytokine based on effect size. The final model included age group (three-level, reference: < 2 years), sex, pre-admission fever ≥ 5 days, wheezing, nasal congestion/rhinorrhea, severe MPP, serum IL-10, and CRP, entered simultaneously.

As this is a retrospective observational study with multiple exploratory comparisons, no correction for multiple testing was applied; reported *P* values should be interpreted as descriptive and hypothesis-generating. Complete-case analysis was used; variables with > 20% missingness were excluded from the multivariable model.

## 3 Results

### 3.1 Viral co-infection profile, age distribution, and quarterly trends of MPP

**Figure 1:**
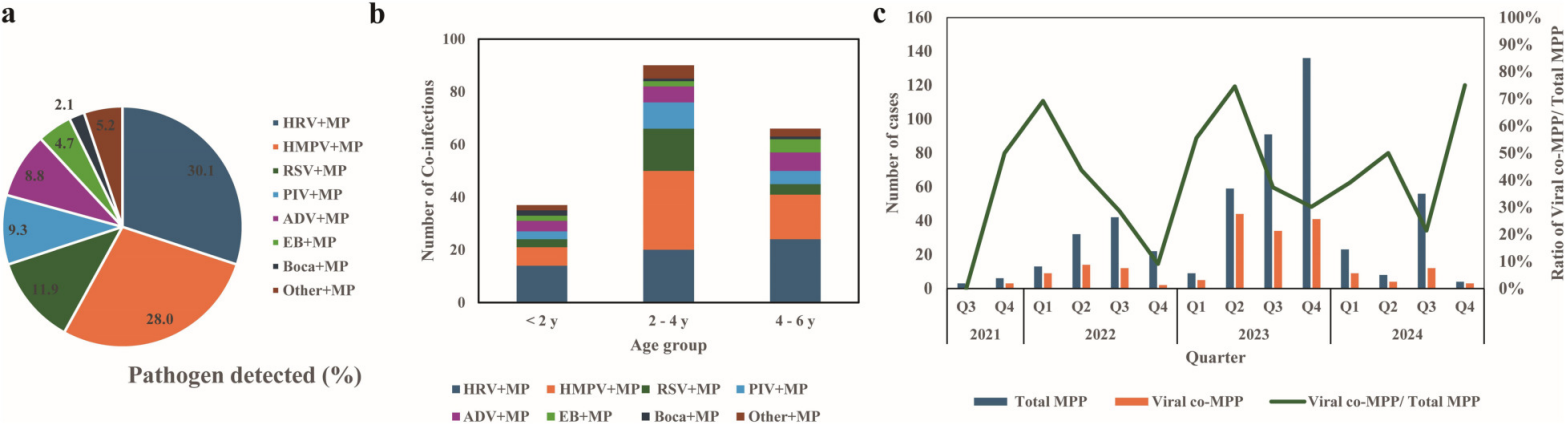
Distribution and temporal trends of respiratory pathogen co-detection among hospitalized preschool children (aged ≤6 years) with Mycoplasma pneumoniae pneumonia (MPP) at the Maternal and Child Health Hospital of Hubei Province, Wuhan, China, July 2021–December 2024. **(a)** Pathogen distribution among children with MPP. **(b)** Pathogen distribution by age group. **(c)** Quarterly distribution of overall respiratory pathogen co-detection across the study period. ADV, adenovirus; Boca, bocavirus; EBV, Epstein–Barr virus; HMPV, human metapneumovirus; MP, *Mycoplasma pneumoniae*; MPP, Mycoplasma pneumoniae pneumonia; PIV, parainfluenza virus; RSV, respiratory syncytial virus; HRV, human rhinovirus. Q1: January-March, Q2: April-June, Q3: July-September, and Q4: October-December.

The five most prevalent pathogens were rhinovirus (30.1%), human metapneumovirus (28.0%), respiratory syncytial virus (11.9%), parainfluenza virus (9.3%), and adenovirus (8.8%), and they collectively accounted for 88.1% of all co-detections (Figure 1A). The participants were categorized into three: Group 1 (age of < 2 years, n = 79), Group 2 (2-4 years, n = 191), and Group 3 (4-6 years, n = 234). Co-detections were recorded for 37 (46.8%, 37/79), 90 (47.1%, 90/191), and 66 cases (28.2%, 66/234) in Groups 1, 2, and 3, respectively. Age-stratified analysis revealed distinct epidemiological patterns: the detection rate of HRV was the highest among infants younger than 2 years (*n* = 37) (37.8%, 14/37), followed by HMPV (18.9%, 7/37) and adenovirus (10.8%, 4/37). Similar distribution patterns were observed for the 4–6 years cohort (*n* = 66), and HRV (36.4%, 24/66), HMPV (25.8%, 17/66), and adenovirus (10.6%, 7/66) were still predominant. The 2–4 years age group (*n* = 90) had a distinct virological profile: the prevalence of HMPV infection (33.3%, 30/90) surpassed that of HRV infection (22.2%, 20/90), and the circulation of RSV (17.8%, 16/90) and PIV (11.1%, 10/90) were substantial. Adenovirus maintained a consistent presence across all age groups (6.7–10.8%), while other pathogens collectively accounted for < 5% of infections in each demographic subgroup (Figure 1B).

As illustrated in Figure 1C, the number of MPP cases exhibited fluctuations during the observation period (2021–2024). The peak incidence varied by year: the peak in 2023 was primarily in the fourth quarter (Q4), while that in 2022 occurred in the third quarter (Q3). Generally, MPP cases began to increase in the second or third quarter of each year. Detection levels for both MPP and viral co-detection remained low throughout 2021. Among the years observed, 2023 recorded the highest burden of MPP cases. Notably, the proportion of viral co-detection cases relative to total MPP cases displayed a distinct seasonal pattern independent of the trend in absolute case numbers, with a consistent peak occurring during the first to second quarters (spring) each year.

### 3.2 Comparison of baseline characteristics between children with MPP mono-infection and viral co-infection profiles

Table 1 summarizes the baseline demographic and clinical characteristics of the study cohorts. This study enrolled 504 patients with MPP. Of these, 311 (61.7%) had only MP infection (MP group), while 193 (38.3%) had viral co-infection (MP + V group). Pediatric patients with MPP and viral co-infection were significantly younger than those with MPP alone (median age: 36.0 vs. 48.0 months; *P* < 0.001). They also had higher rates of wheezing (22.3% vs.10.6%; *P* < 0.001), rhinorrhea/nasal congestion (50.3 vs. 27.0%; *P* < 0.001), and vomiting (20.7% vs. 13.2%; *P* = 0.025).

**Table 1:** Demographic and clinical characteristics of children with MPP stratified by viral co-infection status (2021-2024).

Characteristics	Total (*n* = 504)	MP Mono-infection (*n* = 311)	Viral Co-infection (*n* = 193)	*P-*value
Demographics				
Male sex	287 (56.9%)	184 (59.2%)	103 (53.4%)	0.201
Age (months)	36.0 (24.0–48.0)	48.0 (24.0–48.0)	36.0 (24.0–48.0)	<0.001
Age group:				
<2 years	79 (15.7%)	42 (13.5%)	37 (19.2%)	<0.001
2–4 years	191 (37.9%)	101 (32.5%)	90 (46.6%)	
4–6 years	234 (46.4%)	168 (54.0%)	66 (34.2%)	
Clinical features				
Systemic symptoms				
Fever	458 (90.9%)	280 (90.0%)	178 (92.2%)	0.405
Peak temperature ( °C)	38.8 (38.0–39.3)	38.9 (38.0–39.0)	38.7 (38.0–39.3)	0.589
Lethargy	8 (1.6%)	5 (1.6%)	3 (1.6%)	1.000
Respiratory symptoms				
Cough	499 (99.0%)	307 (98.7%)	192 (99.5%)	0.398
Wheezing	76 (15.1%)	33 (10.6%)	43 (22.3%)	<0.001
Nasal congestion/rhinorrhea	181 (35.9%)	84 (27.0%)	97 (50.3%)	<0.001
Moist rales	147 (29.3%)	90 (29.1%)	57 (29.5%)	0.922
Gastrointestinal symptoms				
Vomiting	81 (16.1%)	41 (13.2%)	40 (20.7%)	0.025
Diarrhea	23 (4.6%)	14 (4.5%)	9 (4.7%)	0.933
Abdominal pain	22 (4.4%)	16 (5.1%)	6 (3.1%)	0.277
Clinical Course & Outcomes				
Hospital stay (days)	6.0 (5.0–8.0)	6.0 (5.0–8.0)	6.0 (5.0–7.0)	0.058
Fever duration pre-admission (days)	4.0 (2.0–6.0)	4.0 (2.8–6.0)	3.0 (2.0–5.0)	<0.001
Cough duration pre-admission (days)	5.0 (3.0–7.0)	5.0 (4.0–7.0)	5.0 (3.0–7.0)	0.033
Symptom onset-to-admission (days)	6.0 (4.0–7.0)	6.0 (4.0–9.0)	5.0 (4.0–7.0)	<0.001
Complications				
Lung consolidation	29 (5.8%)	21 (6.8%)	8 (4.1%)	0.222
Severe pneumonia	61 (12.1%)	31 (10.0%)	30 (15.5%)	0.062
Sepsis	15 (3.0%)	8 (2.6%)	7 (3.6%)	0.498
Extrapulmonary manifestations ^a^	22 (4.4%)	13 (4.2%)	9 (4.7%)	0.796

Extrapulmonary manifestations ^a^ including generalized skin rashes (13, 2.6%), Acute mesenteric lymphadenitis (3, 0.6%), Myocardial injury (5, 1.0%), Elevated liver enzymes (4, 0.8%), and Central nervous system involvement (3, 0.6%). Data are presented as *n* (%) or median (interquartile range, IQR).

Several clinical time intervals were significantly shorter in the MPP co-infection group than in the MPP-only group, including fever duration pre-admission [3.0 (2.0–5.0) vs. 4.0 (2.8–6.0) days; *P* < 0.001], cough duration pre-admission [5.0 (3.0–7.0) vs. 5.0 (4.0–7.0) days; *P* = 0.033], and the time from symptom onset to admission [5.0 (4.0–7.0) vs. 6.0 (4.0–9.0) days; *P* < 0.001]. No statistically significant differences were observed between the two groups regarding peak body temperature, proportion of patients with pulmonary crackles, disease severity, extrapulmonary complications, or length of hospital stay (*P* > 0.05).

### 3.3 Comparison of inflammatory markers between MP mono-infection and viral co-infection groups

As presented in Table 2, children with MP and viral co-infection exhibited significantly elevated serum levels of interleukin-10 (IL-10) [9.15 (5.99–15.04) vs. 7.20 (5.14–10.14) pg/mL; *P* < 0.001], tumor necrosis factor-alpha (TNF-α) [3.47 (2.24–6.67) vs. 3.03 (2.01–5.18) pg/mL; *P* = 0.045], and interferon-gamma (IFN-*γ*) [3.27 (2.19–5.38) vs. 2.81 (1.85–4.70) pg/mL; *P* = 0.033]. Additionally, a reduced level of C-reactive protein (CRP) was observed in this group compared to those with MP mono-infection, with values of 5.45 (1.86–11.88) vs. 6.98 (3.17–14.12) mg/L; *P* = 0.008. No statistically significant differences were noted between the two groups regarding other inflammatory markers, including white blood cell (WBC) count, neutrophil count, lymphocyte count.

**Table 2:** Comparison of inflammatory markers between children with viral co-infection and mono-infection with MPP.

Inflammatory markers	Total (*n* = 504)	MP Mono-infection (*n* = 311)	Viral Co-infection (*n* = 193)	*P* -value
WBC (×10⁹/L)	7.58 (6.07–9.33)	7.52 (6.10–9.20)	7.64 (5.90–9.60)	0.678
Neutrophils (×10⁹/L)	4.15 (3.02–5.47)	4.22 (3.18–5.27)	4.02 (2.80–5.87)	0.584
Lymphocytes (×10⁹/L)	2.48 (1.85–3.34)	2.49 (1.93–3.27)	2.47 (1.76–3.41)	0.808
CRP (mg/L)	6.51 (2.59–13.27)	6.98 (3.17–14.12)	5.45 (1.86–11.88)	0.008
IL-10 (pg/mL)	7.83 (5.25–11.82)	7.20 (5.14–10.14)	9.15 (5.99–15.04)	<0.001
TNF-α (pg/mL)	3.25 (2.06–5.49)	3.03 (2.01–5.18)	3.47 (2.24–6.67)	0.045
IFN-*γ* (pg/mL)	2.92 (1.99–4.97)	2.81 (1.85–4.70)	3.27 (2.19–5.38)	0.033

CRP, C-reactive protein; IFN, interferon; IL, interleukin; n, number of patients; NLR, neutrophil-to-lymphocyte ratio; TNF, tumor necrosis factor; WBC, white blood cell. The data are presented as median (interquartile range, IQR).

### 3.4 Treatment comparison of single-pathogen and co-infection profiles for MPP

As shown in Table 3, The use of systemic glucocorticoids (24.9% vs. 45.5%; *P* < 0.001) and combination therapy with third-generation cephalosporins and macrolides (43.0% vs. 55.0%; *P* = 0.009) was significantly less frequent in the co-infection group. No statistically significant differences were observed between the two groups in the rates of bronchoscopy, overall use of combined antibiotic therapy, or administration of macrolides, quinolones, or tetracyclines (*P* > 0.05).

**Table 3:** Comparison of treatment strategies between viral co-infection and mono-infection in children with MPP. .

Treatments and Procedures	Total (*n* = 504)	MP Mono-infection (*n* = 311)	Viral Co-infection (*n* = 193)	*P-*value
Glucocorticoids	189 (37.6%)	141 (45.5%)	48 (24.9%)	<0.001
BAL	93 (18.5%)	65 (20.9%)	28 (14.5%)	0.072
Antibiotics				
Combined antibiotics	419 (83.1%)	259 (83.3%)	160 (82.9%)	0.912
Macrolides	474 (94.0%)	297 (95.5%)	177 (91.7%)	0.081
Quinolones	7 (1.4%)	5 (1.6%)	2 (1.0%)	0.888
Tetracyclines	14 (2.8%)	9 (2.9%)	5 (2.6%)	1.000
3GC + Macrolide	254 (50.4%)	171 (55.0%)	83 (43.0%)	0.009

BAL, bronchoalveolar lavage; 3GC, third-generation cephalosporin. Data are presented as *n* (%).

### 3.5 Comparison of clinical characteristics of co-infection and mono-infection across age groups

#### 3.5.1 Age-stratified analysis: < 2 years

**Figure 2:**
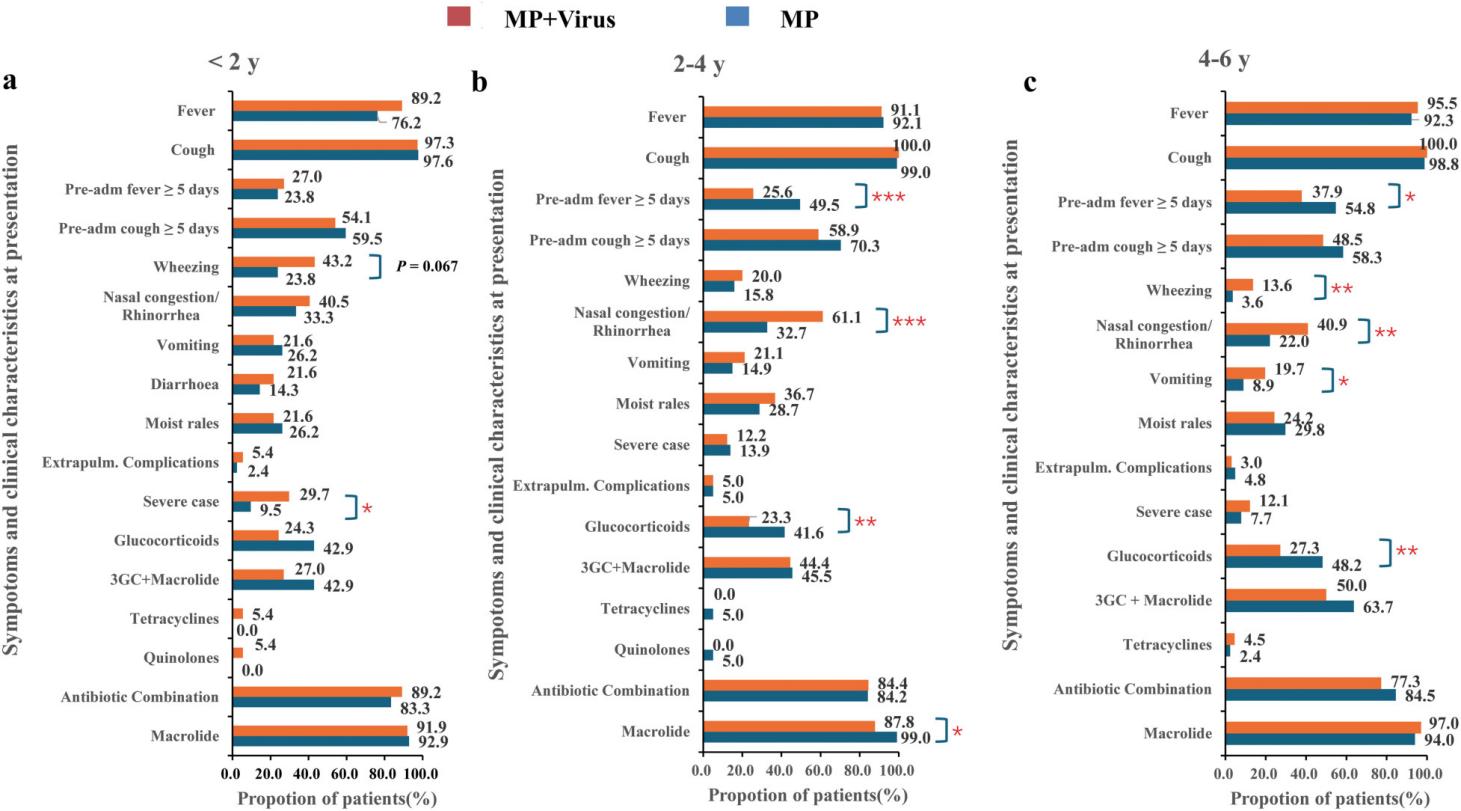
Age-stratified comparison of clinical signs, symptoms, and antibiotic use between hospitalized preschool children with *Mycoplasma pneumoniae* pneumonia (MPP) alone and those with concurrent respiratory viral co-infection. **(a)** Children aged < 2 years. **(b)** Children aged 2–4 years. **(c)** Children aged 4–6 years. Bar graphs depict the frequency (%) of key signs and symptoms (*Y*-axis) in children with MPP alone (blue) and those with viral co-infection (orange). Significant differences compared to the control group are indicated as *P < 0.05, **P < 0.01, ***P < 0.001. MPP, Mycoplasma pneumoniae pneumonia; 3GC, Third-Generation Cephalosporin.

In children under 2 years, co-detection was associated with a higher rate of severe disease (29.7% vs. 9.5%; *P* = 0.046). A case-level review of the 15 severe cases (11 co-infected, 4 mono-infected) identified pulmonary features comprising supplemental oxygen requirement (n = 2), atelectasis or lobar consolidation (n = 3), endobronchial lesions on flexible bronchoscopy (n = 4), and wheezing with respiratory distress (n = 6), together with systemic and extrapulmonary features comprising severe systemic inflammatory response requiring broad-spectrum antimicrobial therapy (n = 2), cardiac enzyme elevation with or without ECG abnormalities (n = 4), transaminase elevation (n = 3), diarrhea with dehydration requiring intravenous rehydration (n = 3), and neutropenia (n = 3). Between-group differences in wheezing (43.2% vs. 23.8%; *P* = 0.067) and systemic corticosteroid use (24.3% vs. 42.9%; *P* = 0.083) did not reach statistical significance (Figure 2A).

#### 3.5.2 Age-stratified analysis: 2–6 years

In children aged 2-6 years (Figure 2B, 2C), severe pneumonia and extrapulmonary complication rates did not differ between groups in either subgroup (all *P* > 0.05). Prolonged pre-admission fever (≥ 5 days) was less frequent with co-detection in both subgroups (2–4 y: 25.6% vs. 49.5%, *P* < 0.001; 4–6 y: 37.9% vs. 54.8%, *P* = 0.020), while rhinorrhea was more frequent (2-4 y: 61.1% vs. 32.7%, *P* < 0.001; 4–6 y: 40.9% vs. 22.0%, *P* = 0.004). Vomiting (19.7% vs. 8.9%; *P* = 0.022) and wheezing (13.6% vs. 3.6%; *P* = 0.005) were more frequent with co-detection only at 4 – 6 y. Systemic corticosteroid use was lower with co-detection in both subgroups (2–4 y: 23.3% vs. 41.6%, *P* = 0.007; 4-6 y: 27.3% vs. 48.2%, *P* = 0.003); macrolide use was lower at 2–4 y (87.8% vs. 99.0%; *P* = 0.001). Other findings did not differ significantly (all *P* > 0.05).

### 3.6 Comparison of MPP Co-infections with HRV, HMPV, and RSV

#### 3.6.1 Clinical characteristics

The time from symptom onset to hospital admission was significantly shorter in both the HMPV [5.0 (4.0–6.0) days] and RSV [4.0 (3.0–5.0) days] groups compared to the HRV group [7.0 (5.0–10.0) days; both *P* = 0.001]. Similarly, the duration of cough prior to admission was significantly shorter in both co-infection groups [HMPV: 4.0 (3.0–5.3) days, *P* = 0.001; RSV: 3.5 (3.0–5.0) days, *P* = 0.001] than in the HRV group [6.0 (3.0–10.0) days]. Furthermore, fever was universally present in both the HMPV (100% vs. 86.2%; *P* = 0.014) and RSV (100% vs. 86.2%; *P* = 0.143) cohorts compared to the HRV group (Table 4). The proportions of patients with extrapulmonary complications or severe disease did not differ significantly among the groups (Table 4).

**Table 4:** Comparison of the clinical characteristics, inflammatory markers, and treatment strategies in patients co-infected with HRV, HMPV, RSV, and MP.

Variable	HRV with MPP (*n* = 57)	HMPV with MPP (*n* = 54)	*P*-value	RSV with MPP (*n* = 23)	*P*-value
Demographics					
Gender, male	30 (51.7%)	24 (44.4%)	0.456	11 (47.8%)	0.809
Age (months)	36 (24–48)	36 (24–48)	0.971	24 (24–36)	0.304
Clinical features					
Maximum temperature ( °C)	38.5 (37.4–39.2)	39.0 (38.6–39.6)	0.001	38.8 (38.3–39.2)	0.142
Fever	50 (86.2%)	54 (100.0%)	0.014	23 (100.0%)	0.143
Wheezing	15 (25.9%)	11 (20.4%)	0.512	4 (17.4%)	0.564
Nasal congestion/rhinorrhea	25 (43.1%)	36 (66.7%)	0.014	16 (69.6%)	0.048
Chills	3 (5.2%)	10 (18.5%)	0.038	3 (13.0%)	0.345
Vomiting	6 (10.3%)	19 (35.2%)	0.003	7 (30.4%)	0.042
Extrapulmonary complications	2 (3.4%)	1 (1.9%)	1.000	2 (8.7%)	0.679
Moist rales	13 (22.4%)	16 (29.6%)	0.398	12 (52.2%)	0.015
Clinical course					
Hospital stay (days)	6.0 (5.0–7.0)	6.0 (5.0–7.0)	0.093	6.0 (5.0–7.0)	0.440
Fever duration pre-admission (days)	2.0 (1.0–5.0)	4.0 (3.0–5.0)	0.020	3.0 (2.5–4.0)	0.422
Cough duration before admission (days)	6.0 (3.0–10.0)	4.0 (3.0–5.3)	0.001	3.5 (3.0–5.0)	0.001
Time to onset (days)	7.0 (5.0–10.0)	5.0 (4.0–6.0)	0.001	4.0 (3.0–5.0)	0.001
Severe disease	8 (13.8%)	8 (14.8%)	1.000	3 (13.0%)	1.000
Inflammatory markers					
WBC (×10⁹/L)	7.19 (4.92–12.04)	6.51 (5.00–9.12)	0.004	8.30 (7.36–10.02)	0.679
Neutrophils (×10⁹/L)	3.93 (2.69–4.06)	3.05 (2.46–4.69)	0.032	5.76 (4.11–7.36)	0.033
Lymphocytes (×10⁹/L)	2.70 (1.72–7.31)	2.25 (1.73–3.05)	0.013	2.00 (1.64–2.72)	0.005
NLR	1.17 (0.62–2.13)	1.43 (0.91–2.16)	0.907	2.87 (1.57–4.30)	0.003
CRP (mg/L)	2.65 (0.94–14.87)	1.86 (1.25–8.73)	0.082	7.28 (2.97–15.27)	0.312
IL-10 (pg/mL)	5.00 (2.97–5.95)	8.76 (5.58–18.66)	0.037	10.97 (7.74–20.23)	0.024
IFN-γ (pg/mL)	2.24 (1.12–2.77)	4.34 (2.68–5.96)	0.028	3.98 (2.41–4.88)	0.129
Treatment					
Glucocorticoids	20 (34.5%)	4 (7.4%)	<0.001	5 (21.7%)	0.300
BAL	11 (19.0%)	3 (5.6%)	0.045	0 (0.0%)	0.029
Combined antibiotics	45 (77.6%)	48 (88.9%)	0.111	21 (91.3%)	0.264
Macrolides	55 (94.8%)	46 (85.2%)	0.163	20 (87.0%)	0.454

BAL, bronchoalveolar lavage; CRP, C-reactive protein; HMPV, human metapneumovirus; HRV, human rhinovirus; IFN, interferon; IL, interleukin; MPP, Mycoplasma pneumoniae pneumonia; NLR, neutrophil-to-lymphocyte ratio; RSV, respiratory syncytial virus. *P*-values represent comparisons between the HRV group and the HMPV group (first *P* column) and between the HRV group and the RSV group (second *P* column). Data are presented as *n* (%) or median (interquartile range, IQR).

#### 3.6.2 Group-specific clinical manifestations

The HMPV group exhibited a longer fever duration pre-admission [4.0 (3.0–5.0) vs. 2.0 (1.0–5.0) days; *P* = 0.020], a higher maximum body temperature [39.0 (38.6–39.6) vs. 38.5 (37.4–39.2) °C; *P* = 0.001], and a higher frequency of systemic symptoms including nasal congestion/rhinorrhea (66.7% vs. 43.1%; *P* = 0.014), vomiting (35.2% vs. 10.3%; *P* = 0.003), and chills (18.5% vs. 5.2%; *P* = 0.038) than the HRV group (Table 4). The RSV group showed a higher incidence of moist rales (52.2% vs. 22.4%; *P* = 0.015), vomiting (30.4% vs. 10.3%; *P* = 0.042), and nasal congestion/rhinorrhea (69.6% vs. 43.1%; *P* = 0.048) than the HRV group (Table 4).

#### 3.6.3 Inflammatory and immunological profiles

HMPV co-infection was associated with significantly lower leukocyte counts (WBC: 6.51 (5.00–9.12) vs. 7.19 (4.92–12.04) × 10⁹/L, *P* = 0.004; neutrophils: 3.05 (2.46–4.69) vs. 3.93 (2.69–4.06) × 10⁹/L, *P* = 0.032; lymphocytes: 2.25 (1.73–3.05) vs. 2.70 (1.72–7.31) × 10⁹/L, *P* = 0.013) but elevated cytokine levels (IL-10: 8.76 (5.58–18.66) vs. 5.00 (2.97–5.95) pg/mL, *P* = 0.037; IFN-*γ*: 4.34 (2.68–5.96) vs. 2.24 (1.12–2.77) pg/mL, *P* = 0.028) compared to HRV co-infection.

RSV co-infection was characterized by a heightened neutrophilic response, evidenced by significantly higher neutrophil counts [5.76 (4.11–7.36) vs. 3.93 (2.69–4.06) × 10⁹/L, *P* = 0.033] and an elevated neutrophil-to-lymphocyte ratio [2.87 (1.57–4.30) vs. 1.17 (0.62–2.13), *P* = 0.003], along with increased IL-10 concentrations [10.97 (7.74–20.23) vs. 5.00 (2.97–5.95) pg/mL, *P* = 0.024] (Table 4).

#### 3.6.4 Treatment patterns

Bronchoscopy was performed significantly less frequently in the RSV group (0.0% vs. 19.0%; *P* = 0.029) and showed a trend towards less use in the HMPV group (5.6% vs. 19.0%; *P* = 0.045). Systemic corticosteroids were administered significantly less often in the HMPV group (7.4% vs. 34.5%; *P* < 0.001), but no significant difference was observed between the RSV and HRV groups (21.7% vs. 34.5%; *P* = 0.300) (Table 4). The utilization of combination antibiotic therapy did not show a significant difference between the HMPV and HRV groups, nor between the RSV and HRV groups (HMPV: 88.9% vs. 77.6%; *P* = 0.111; RSV: 91.3% vs. 77.6%; *P* = 0.264).

### 3.7 Factors associated with respiratory virus coinfection in children with MP pneumonia

After additional adjustment for fever duration, respiratory viral coinfection was significantly associated with a higher incidence of wheezing [adjusted odds ratio (aOR) = 1.909; 95% confidence interval (CI): 1.131–3.222] and nasal obstruction/rhinorrhea (aOR=2.268; 95% CI: 1.532–3.359), as well as more severe manifestations of pneumonia (aOR=1.797; 95% CI: 1.019–3.169). Additionally, elevated serum IL-10 levels (aOR=1.038; 95% CI: 1.015–1.062) were independently associated with viral coinfection.

In contrast, fever lasting ≥5 days before admission (aOR=0.475; 95% CI: 0.321–0.703) was inversely associated with viral coinfection. Children aged 4–6 years had a significantly lower risk of viral coinfection compared to those under 2 years old (aOR=0.528; 95% CI: 0.308–0.903) (Table 5).

**Table 5:** Factors associated with respiratory virus co-infection in children with MP pneumonia. .

Variable	OR (95% CI)	*P* Value	aOR* (95% CI)	*P* Value
Age group				
2–4 years vs. < 2 years	1.012 (0.598–1.711)	0.996	1.120 (0.656–1.913)	0.678
4–6 years vs. < 2 years	**0.446 (0.264–0.754)**	**0.003**	**0.528 (0.308–0.903)**	**0.020**
Fever before admission ≥5 day	**0.449 (0.307–0.657)**	**<0.001**	**0.475 (0.321–0.703)**	**<0.001**
Wheezing	**2.415 (1.472–3.962)**	**<0.001**	**1.909 (1.131–3.222)**	**0.015**
Nasal obstruction/Rhinorrhea	**2.731 (1.873–3.982)**	**<0.001**	**2.268 (1.532–3.359)**	**<0.001**
Severe pneumonia	1.662 (0.971–2.846)	0.064	**1.797 (1.019–3.169)**	**0.043**
IL-10, pg/mL	**1.044 (1.020–1.068)**	**<0.001**	**1.038 (1.015–1.062)**	**0.001**
CRP, mg/dL	0.992 (0.977–1.007)	0.273	0.994 (0.979–1.009)	0.406

Bold values denote statistical significance. aOR, adjusted odds ratio; CI, confidence interval; IL, interleukin; CRP, C-reactive protein.

*Adjusted for age, sex, and fever before admission ≥ 5 day.

### 3.8 Impact of delayed treatment

As illustrated in Table 6, utilizing fever lasting ≥ 5 days prior to admission as an indicator of delayed treatment, the Delayed group (*n* = 210) exhibited a clinical profile that was markedly different from that of the Non-delayed group (*n* = 294). The Delayed group consisted of significantly older children (median 48 vs. 36 months; *P* < 0.001). Furthermore, this group demonstrated significantly higher rates of severe pneumonia (15.7% vs. 9.5%; *P* = 0.036). They were also more likely to receive combination therapy with third-generation cephalosporin and macrolide (58.6% vs. 44.6%; *P* = 0.002) and undergo bronchoalveolar lavage (24.3% vs. 14.3%; *P* = 0.004). In contrast, the Non-delayed group had higher overall viral co-infection rates (45.9% vs. 27.6%; *P* < 0.001), including significantly more HMPV (13.3% vs. 7.1%; *P* = 0.028) and RSV infections (6.8% vs. 1.4%; *P* = 0.008).

**Table 6:** Characteristics and outcomes of children with MPP stratified by treatment timing.

Characteristic	Delayed Treatment (≥ 5 days fever) (*n* = 210)	Non-Delayed Treatment (< 5 days fever) (*n* = 294)	*P*-value
Gender, male	109 (51.9%)	178 (60.5%)	0.053
Age (years)	48 (36–60)	36 (24–48)	<0.001
Overall viral co-infection	58 (27.6%)	135 (45.9%)	<0.001
hMPV co-infection	15 (7.1%)	39 (13.3%)	0.028
RSV co-infection	3 (1.4%)	20 (6.8%)	0.008
HRV co-infection	19 (9.0%)	38 (12.9%)	0.140
Onset-to-admission time (days)	6.0 (5.0–7.25)	4.5 (3.0–7.0)	<0.001
Severe case	33 (15.7%)	28 (9.5%)	0.036
Hospital stay length (days)	6.0 (6.0–8.0)	6.0 (5.0–8.0)	0.053
Combination antibiotics	174 (82.9%)	245 (83.3%)	0.888
3GC + Macrolide	123 (58.6%)	131 (44.6%)	0.002
Bronchoscopy	51 (24.3%)	42 (14.3%)	0.004

3GC, third-generation cephalosporin; hMPV, human metapneumovirus; HRV, human rhinovirus; RSV, respiratory syncytial virus. Data are presented as *n* (%) or median (interquartile range, IQR).

## 4 Discussion

Consistent with previous reports (5, 6, 15), MP was detected throughout the year with notable seasonal variations. The detection rate in 2021 was markedly lower, a trend consistent with the implementation of COVID-19 containment measures during that period. Epidemic peaks occurred in the third quarter (Q3) during both 2022 and 2024. In contrast, the 2023 pattern was distinctive, with incidence rising from the second quarter (Q2) and peaking in the fourth quarter (Q4), a trajectory that mirrors the documented global resurgences (7, 22).

Respiratory viral co-detection rates in children with MPP vary across studies (reported range: 27.3%–56.1%) (6, 7, 11, 23, 24). In this study, viral co-detection was common, occurring in 38.3% (193/504) of children with MPP. Respiratory syncytial virus (RSV), rhinovirus (RV), and human metapneumovirus (HMPV) are the predominant respiratory viruses associated with community-acquired pneumonia (CAP) in children (5, 25–27). In this study, RV was the predominant co-infecting pathogen (accounting for 30.1%). It was followed by HMPV (accounting for 28.0%) and RSV (accounting for 11.9%). The high prevalence of human metapneumovirus (HMPV) co-infection observed in this study (28.0%) underscores its significant role as a pediatric pathogen, particularly among children under the age of 5 years (28, 29). A notable observation was the distinct age-specific distribution pattern: the co-detection rate was highest among children aged 2 to 4 years, reaching 47.1% (90/191), which is significantly greater than that of the 4 to 6-year-old group (28.2%, 66/234), and comparable to the younger cohort of children aged 2 years and below (46.8%, 37/79). Within this subgroup (ages 2–4), HMPV emerged as the most prevalent co-infecting pathogen, accounting for 33.3% (30/90) of cases, surpassing rhinovirus (HRV) at a rate of 22.2% (20/90). This age-related predominance is consistent with existing literature indicating that HMPV infection predominantly affects young children, with median ages at onset reported as being approximately 43 months and 33.5 months; additionally, a substantial proportion develops pneumonia (29, 30). Our findings collectively highlight the significant clinical burden that HMPV places on this vulnerable population segment. This underscores the need for increased clinical vigilance and the implementation of targeted early prevention strategies.

Consistent with previous reports (30–32), MPP is primarily characterized by acute respiratory symptoms, including high fever, cough, wheezing, rhinorrhea, and vomiting. Moreover, our study identifies children under 2 years of age as particularly susceptible to severe cases following co-detection, indicating that the impact of viral co-infection is not uniform across age groups. This pattern is consistent with the possibility that it poses a greater challenge to individuals with still-maturing immune function, as suggested by previous literature (11, 33, 34). Furthermore, we demonstrate that the effects of co-detection are pathogen-specific, with different viruses eliciting unique immune and inflammatory responses. Specifically, co-infection with human metapneumovirus (HMPV) triggers a more pronounced Th1/anti-inflammatory response, evidenced by elevated levels of interferon-gamma (IFN-*γ*) and interleukin-10 (IL-10), alongside relatively lower lymphocyte counts.

The elevated IFN-*γ* levels indicate activation of a vigorous Th1-type immune response (35, 36). The subsequent lymphopenia may reflect the migration of activated immune cells to the pulmonary site and the normal apoptosis of effector cells during the later stages of the immune response (37). Collectively, this pattern (IFN-*γ*↑ → lymphocytes↓ → IL-10↑) suggests a sequential transition from a robust antiviral response to an immune regulatory/contraction phase (38–40). The clinical significance of IL-10 in this cohort, however, remains unclear; its role has dual potential, possibly representing a beneficial self-limiting mechanism or being linked to immunosuppression-associated adverse outcomes, as reported in other viral diseases (41). Thus, while this immune signature offers valuable insights into disease mechanisms, its definitive clinical implications await validation in prospective studies.

Some studies indicate that viral co-infection may exacerbate corticosteroid-related adverse outcomes, prolong respiratory insufficiency, and increase mortality and complications, particularly with prolonged administration (42). Corticosteroids are occasionally employed as an adjunctive treatment in cases of viral pneumonia, acute respiratory distress syndrome (ARDS), or severe pneumonia due to their anti-inflammatory properties. However, their efficacy and safety depend on disease severity, inflammatory status, and host immunity (43). In contrast, the “high IL-10 with neutrophilia” pattern in RSV pneumonia presents a distinct scenario, where persistent neutrophilic inflammation coexists with concurrent immunoregulatory signals. This coexistence may indicate a dysregulated inflammatory resolution, potentially underlying a greater clinical reliance on glucocorticoids (44).

Our findings indicate reduced use of systemic glucocorticoids and combination therapy with third-generation cephalosporins and macrolides in co-infected patients. Although not directly assessed in this study, existing literature suggests concerns about macrolide resistance or bacterial co-infections may also contribute to delayed aggressive antibiotic use (45, 46). One plausible explanation is that, given the ineffectiveness of macrolides against viruses and concerns regarding antibiotic resistance, practitioners are exercising greater caution in prescribing them. This revised approach may result in an increased emphasis on supportive care or a more direct transition to alternative antibiotics for patients with a poor initial response.

In this study cohort, hospitalization due to persistent fever lasting more than five days was associated with more severe disease outcomes, including a higher incidence of severe pneumonia, a greater need for bronchoalveolar lavage, and increased use of combination antibiotic therapy. Clinical deterioration in such cases is often accompanied by signs of hypoxemia, such as tachypnea and low oxygen saturation (47, 48). This observed association suggests that delayed hospital admission is an important clinical sign that requires high vigilance in young children.

## 5 Conclusions

In summary, this study demonstrates that viral co-detection in preschool-aged children with MPP is associated with a more severe clinical course, particularly in those under two years of age. Pathogen-specific immune patterns were observed, with HMPV co-detection characterized by a Th1/anti-inflammatory profile (elevated IFN-γ and IL-10 with relative lymphopenia) and RSV co-detection characterized by a neutrophilic pattern with concurrent IL-10 elevation. These findings are hypothesis-generating; the observed associations do not establish causality, nor do they provide direct evidence that altering diagnostic or therapeutic intensity based on viral co-detection status would improve clinical outcomes. In clinical settings where multiplex respiratory panels are already employed, closer monitoring of very young children with MPP and viral co-detection may be warranted, although this should be confirmed in prospective multicenter studies. The age- and pathogen-specific heterogeneity of viral-co-infected pediatric MPP described here may inform future prospective investigations.

## 6 Limitations

This study has several limitations. First, distinguishing active MP infection from asymptomatic colonization remains clinically challenging, and no widely accepted gold standard exists for definitive differentiation. Although MPP was diagnosed comprehensively on the basis of clinical manifestations, imaging, and laboratory findings, PCR-detected MP may represent colonization in some children whose symptoms were primarily driven by co-infecting viruses. Diagnosis relied on molecular detection without paired serological confirmation, and macrolide resistance testing (23S rRNA mutation analysis) was not routinely performed during the study period, precluding evaluation of resistance-related outcomes.

Second, our cohort comprised MPP cases diagnosed via the multiplex PCR panel pathway rather than all MPP admissions. Because this pathway is preferentially used when viral co-infection is clinically suspected, the cohort may have been enriched for cases with overlapping clinical features, potentially limiting generalizability of the observed co-detection rate and between-group differences.

Third, as a single-center retrospective study with modest sample size, our findings, particularly subgroup analyses in children under 2 years (n = 79), had limited statistical power, and no adjustment for multiple comparisons was applied given the exploratory nature of the analyses. Prospective multicenter studies with uniform multiplex testing, paired serological confirmation, and macrolide resistance characterization across diverse populations are needed to validate and extend these findings.

## Data Availability

The original contributions presented in the study are included in the article/Supplementary Material, further inquiries can be directed to the corresponding author/s.
